# Supplementary material for: Reducing sarcolipin expression mitigates Duchenne muscular dystrophy and associated cardiomyopathy in mice
Source: Nat Commun. 2017 Oct 20;8:1068. doi: 10.1038/s41467-017-01146-7 (PMC5648780; doi:10.1038/s41467-017-01146-7)
Supplement: Supplementary file 1 — Supplementary Information [file 41467_2017_1146_MOESM1_ESM.pdf]

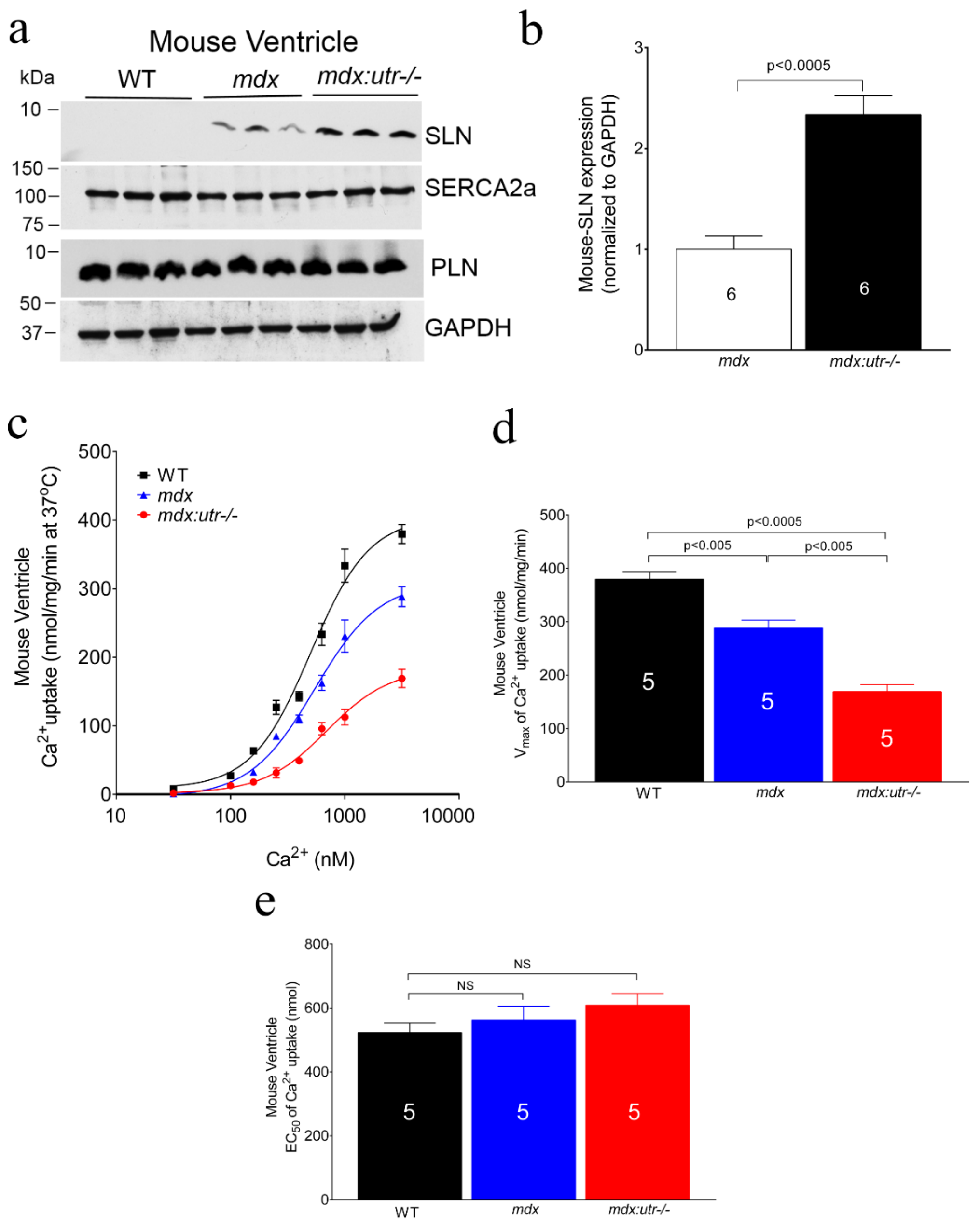

Supplementary Figure 1: SLN upregulation in the ventricles of DMD models. (a) Representative western blots (uncropped) showing SLN upregulation in the ventricles of *mdx* and *mdx:utr-/-* mice. SERCA2a and PLN levels are unchanged. (b) Quantitation show that SLN levels are significantly higher in the ventricles of *mdx:utr-/-* mice compared to that of *mdx* mice. Data are presented as mean ± SEM. The *n* number for each group and the *p* values (*t*-test with Welch's correction) are shown within the graph. (c) The rate of Ca^{2+} dependent SR Ca^{2+} uptake and (d) the V_{max} of Ca^{2+} uptake are significantly reduced in the ventricles of both *mdx* and *mdx:utr-/-* mice. (e) The EC_{50} of Ca^{2+} uptake is unaltered between WT and DMD mice. Data are presented as mean ± SEM. The *n* number for each group and the *p* values (*t*-test with Welch's correction) are shown within the graph. Tissues from 3-4 month old mice are used for all the above experiments.

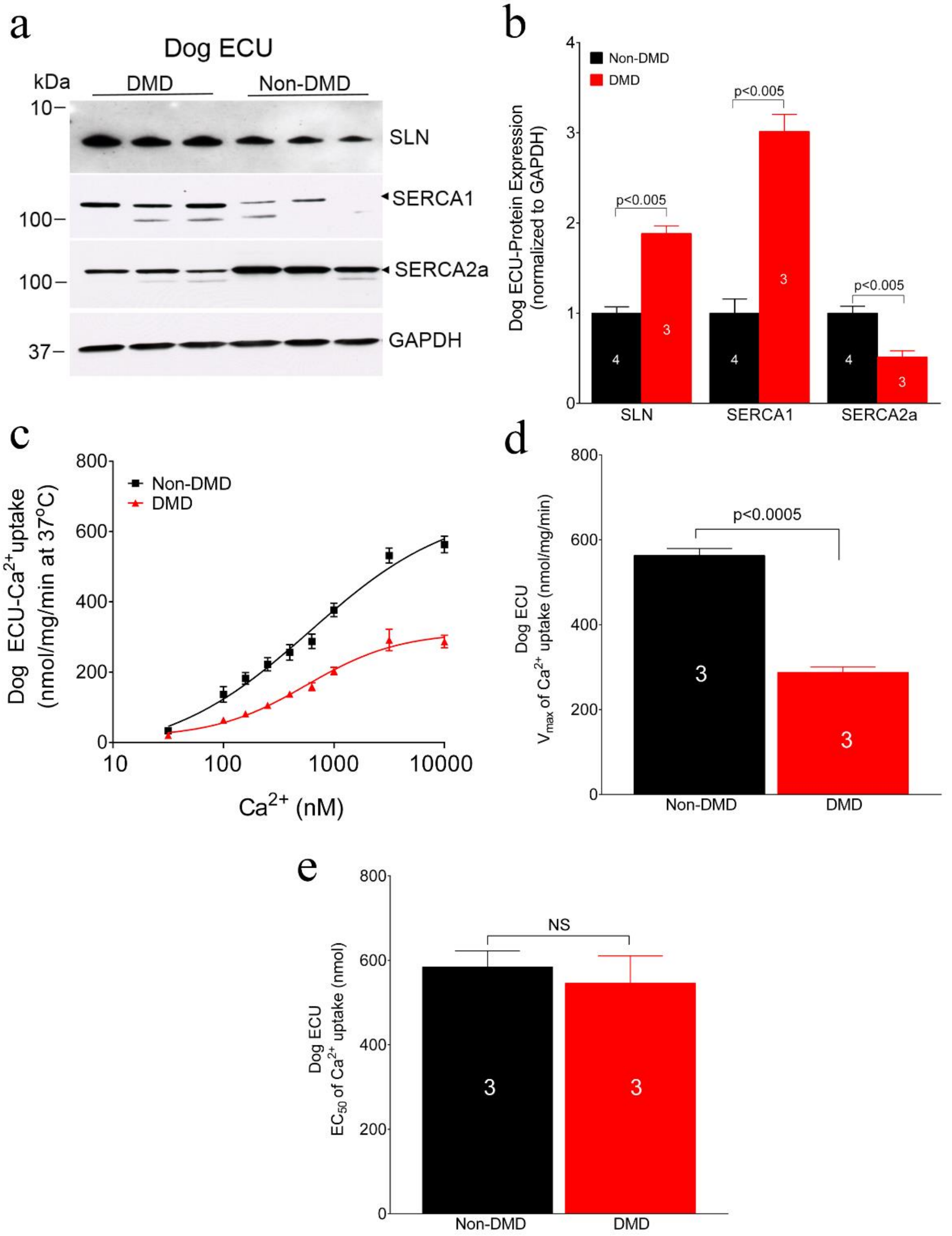

Supplementary Figure 2: SLN upregulation in muscles of DMD dogs. (a) Representative western blots showing the protein levels of SLN and SERCA isoforms in the ECU muscles of DMD and non-DMD dogs. Uncropped scans of the western blots are shown in Supplementary Fig. 9a. (b) Quantitation show that SLN and SERCA1 levels are significantly increased, whereas SERCA2a level is significantly decreased in the ECU of DMD dogs. Data are presented as mean ± SEM. The *n* number for each group and the *p* values (*t*-test with Welch's correction) are shown within the graph. (c) The rate of Ca^{2+} dependent SR Ca^{2+} uptake, and (d) the V_{max} of Ca^{2+} uptake are significantly reduced in the ECU muscles of DMD dogs. (e) The EC_{50} of Ca^{2+} uptake is unaltered between non-DMD and DMD dog tissues. Data are presented as mean ± SEM. The *n* number for each group and the *p* values (*t*-test with Welch's correction) are shown within the graph.

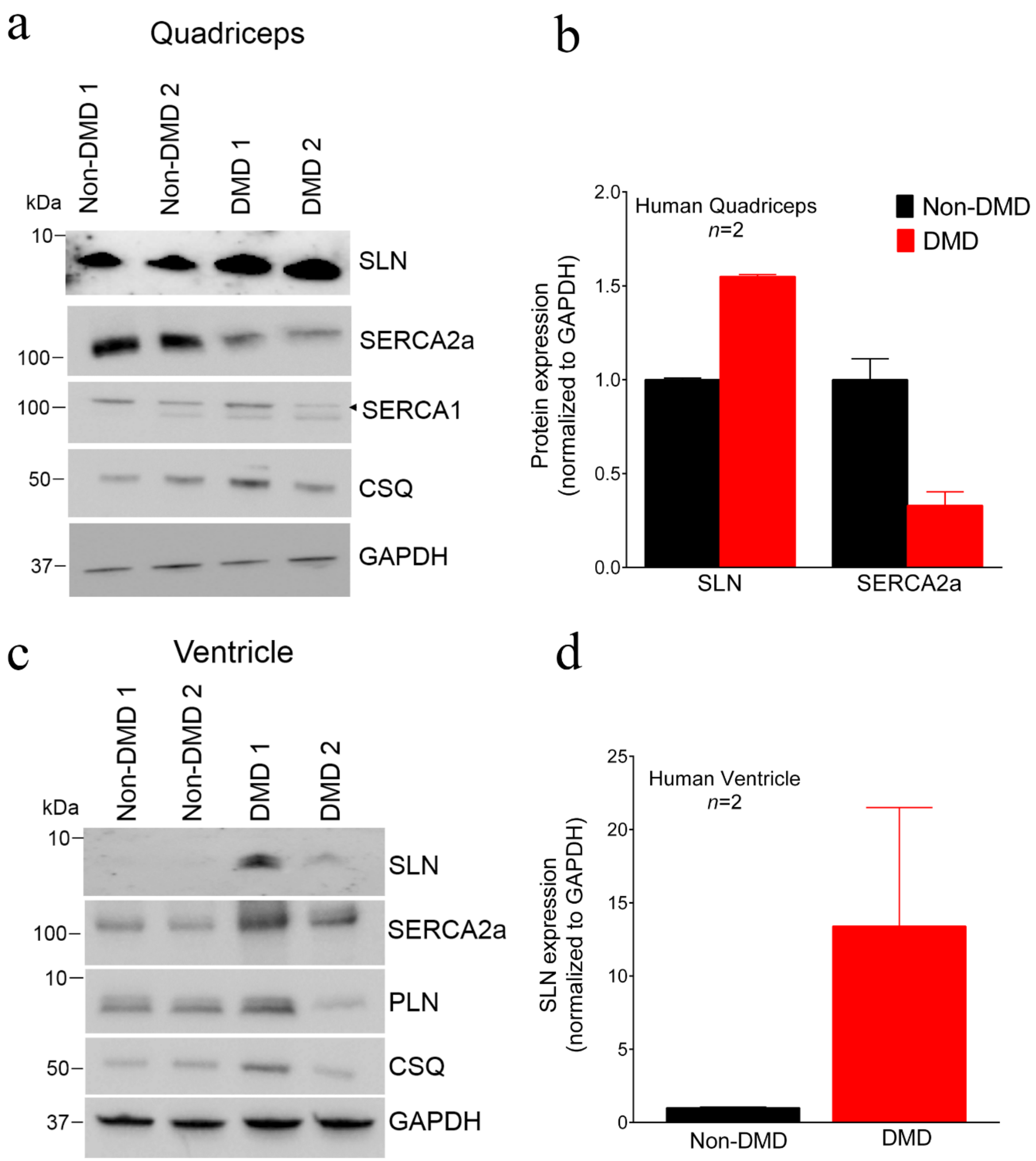

Supplementary Figure 3: SLN upregulation in the heart and muscles of DMD patients. (a) Western blot analysis of SLN, SERCA1, SERCA2a and CSQ protein levels in the quadriceps of DMD patients. (b) Quantitation show that SLN is upregulated ~1.5 fold and SERCA2a is downregulated >50% in the quadriceps of DMD patients compared to that of non-DMD controls, *n*=2 per group. (c) Western blot analysis of SLN, SERCA2a, PLN and CSQ proteins in the human ventricular biopsies. (d) Quantitation show that SLN levels are abnormally high in the ventricular biopsies from DMD patients, *n*=2 per group. Data are presented as mean ± SEM. Uncropped scans of the western blots are shown in Supplementary Fig. 9b-9c.

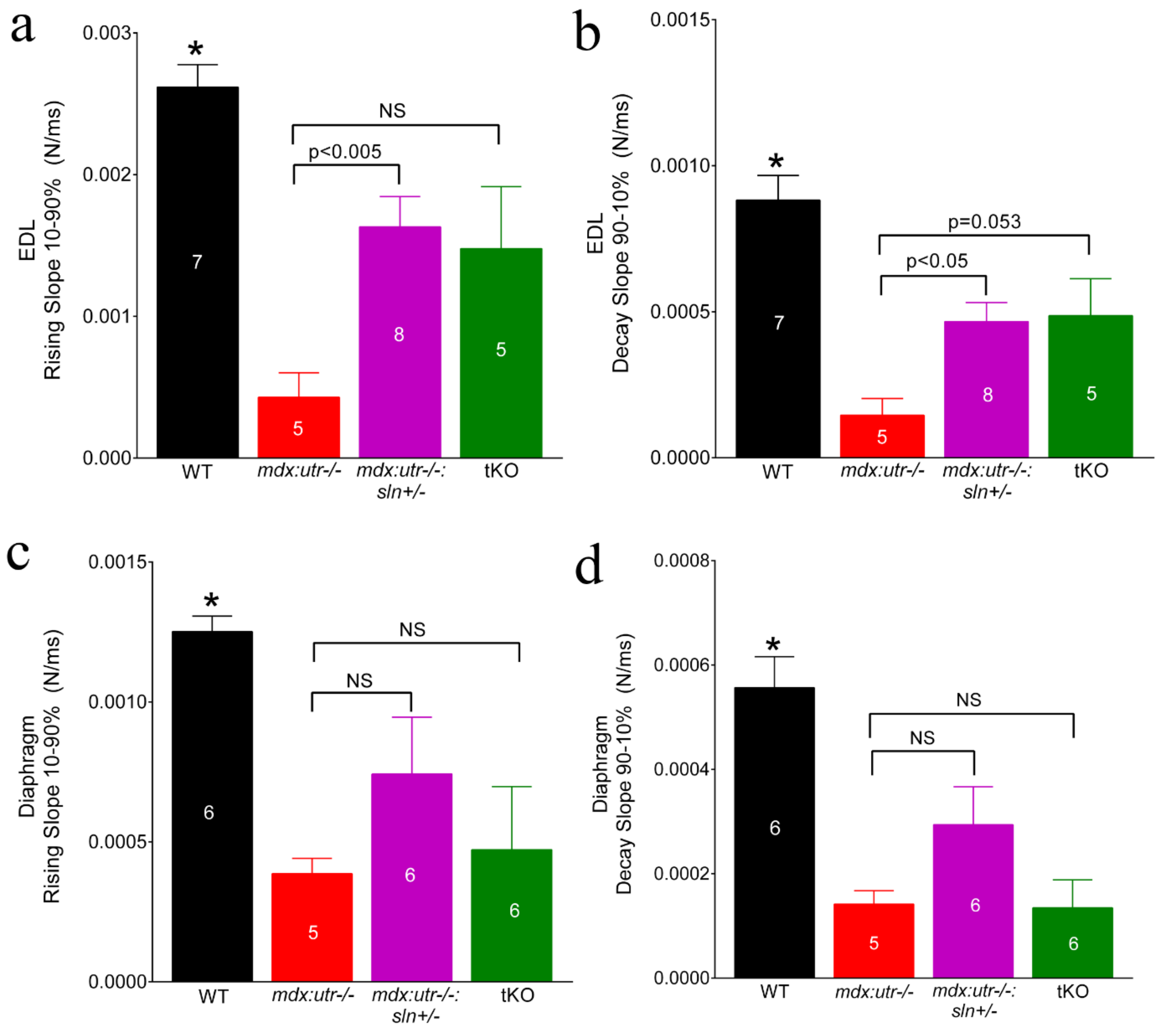

Supplementary Figure 4: Reduction in SLN expression improves muscle contraction. (a, b) The EDL muscles from *mdx:utr-/-:sln+/-* mice exhibits increased rate of contraction as represented by 10%-90% rising slope and increased rate of relaxation as represented by 90%-10% decay slope at 2 Hz in comparison with that of *mdx:utr-/-* mice. (c, d) These changes were less prominent in the diaphragm of *mdx:utr-/-:sln+/-* mice. Data are presented as mean ± SEM. The *n* number for each group and the *p* values (*t*-test with Welch's correction) are shown within the graph. *significantly different from other groups (p<0.05).

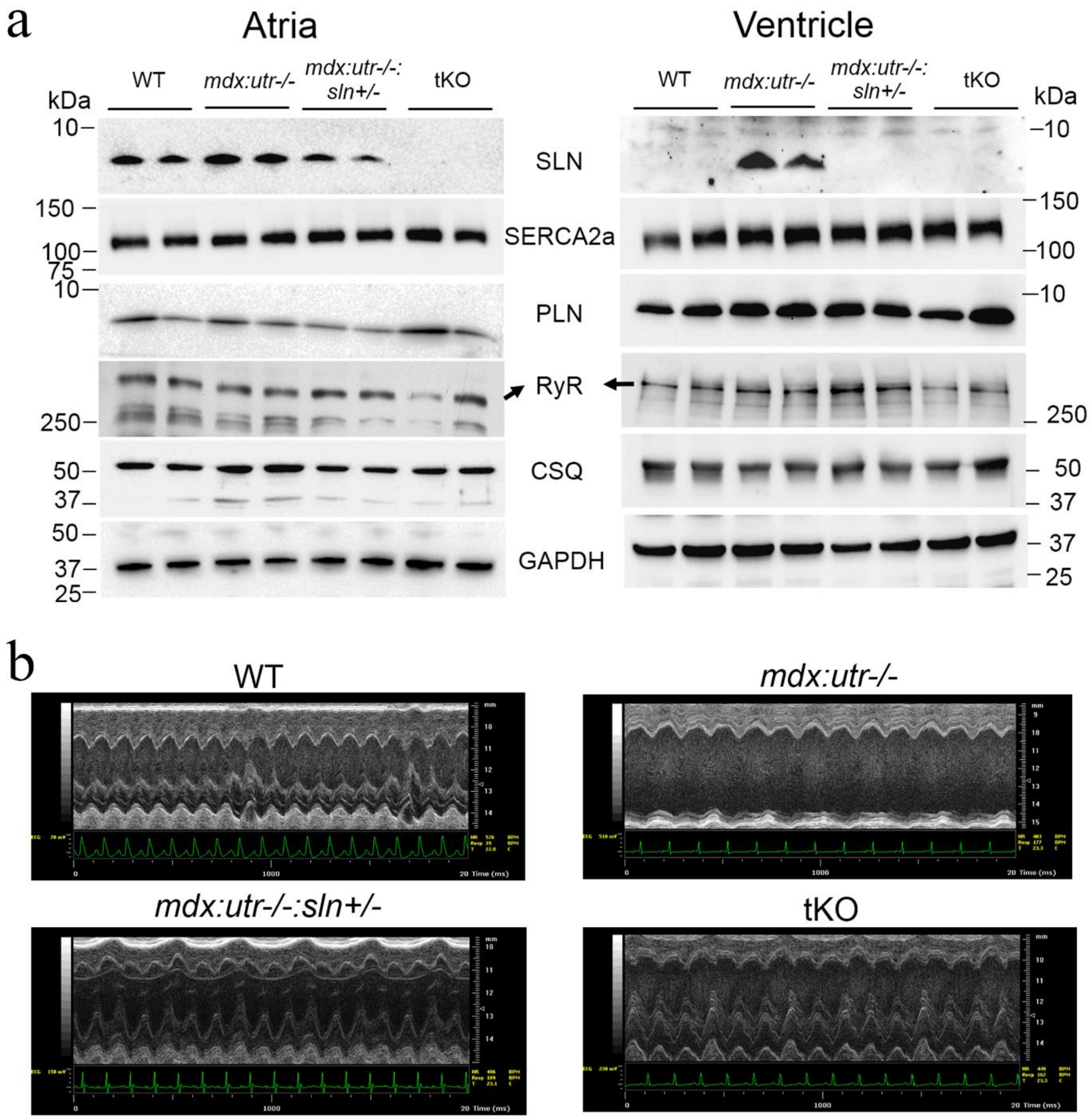

Supplementary Figure 5: Reducing SLN expression improves cardiac function in DMD. (a) Representative western blots (uncropped) showing SERCA2a, SLN, PLN, CSQ and RyR protein levels in atria and in the ventricles of WT, *mdx:utr-/-*, *mdx:utr-/-:sln+/-* and tKO mice. (b) Representative M-mode echocardiography images of left ventricles in 3-4 month old WT, *mdx:utr-/-*, *mdx:utr-/-:sln+/-* and tKO mice at baseline.

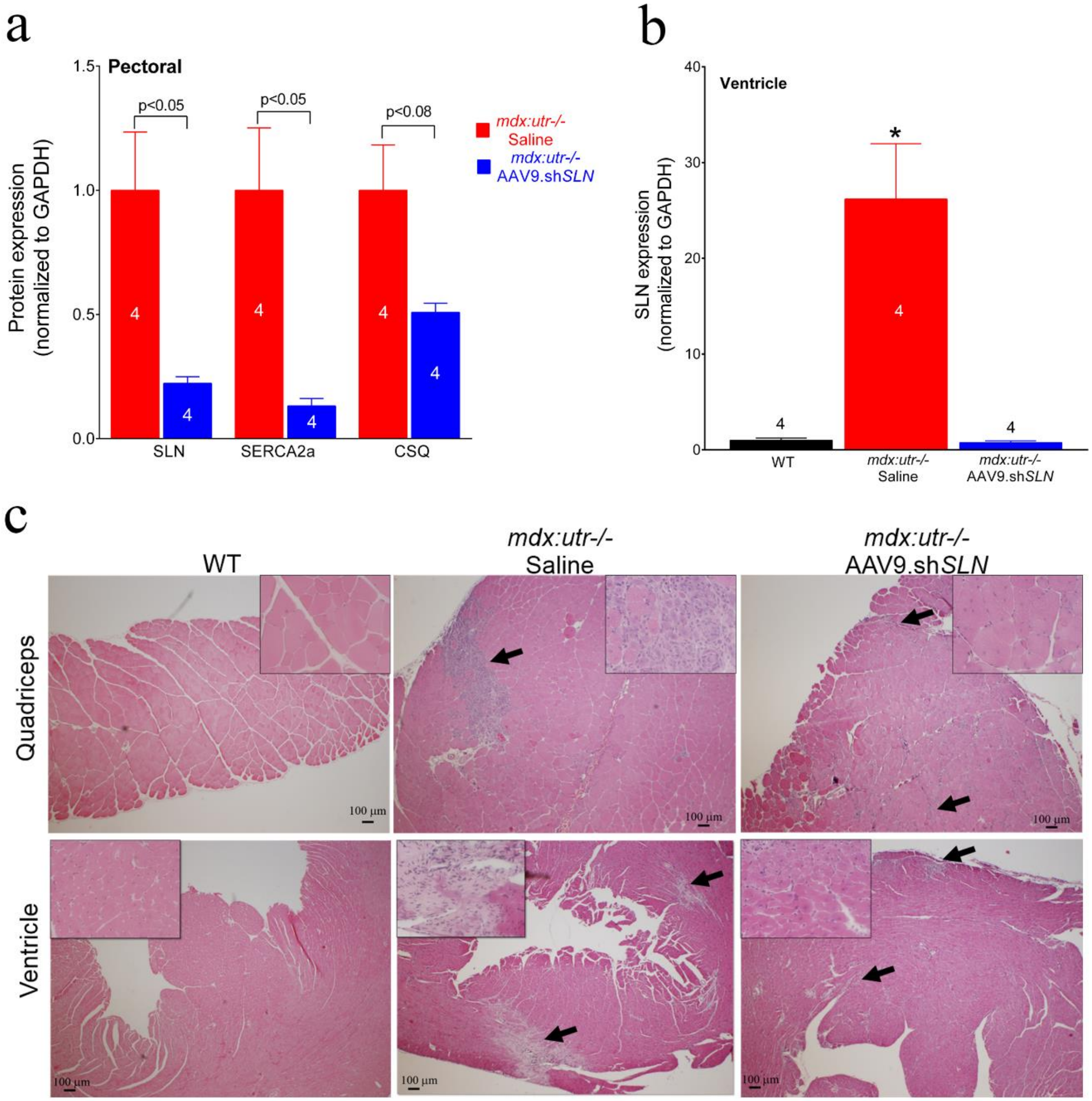

Supplementary Figure 6: AAV9.sh*SLN* treatment mitigates DMD in mice. (a, b) Quantitation of signals from western blots (Fig. 6a-6b) show that AAV treatment significantly reduced SLN protein expression in both skeletal (pectorals) and ventricles of *mdx:utr-/-* mice. In addition, AAV treatment suppresses the induction of SERCA2a and CSQ expression in the pectorals of *mdx:utr-/-* mice. Data are presented as mean ± SEM. *significantly different from other groups (*n*=4 per group, p<0.05, *t*-test with Welch's correction). (c) Representative H&E staining of quadriceps and ventricular sections show decreased mononuclear infiltration in AAV treated groups. Original magnification is 5X. Inset is 40X focusing the necrotic areas. Arrow indicates the necrotic areas with mononuclear infiltration. Scale bar is 100 μm.

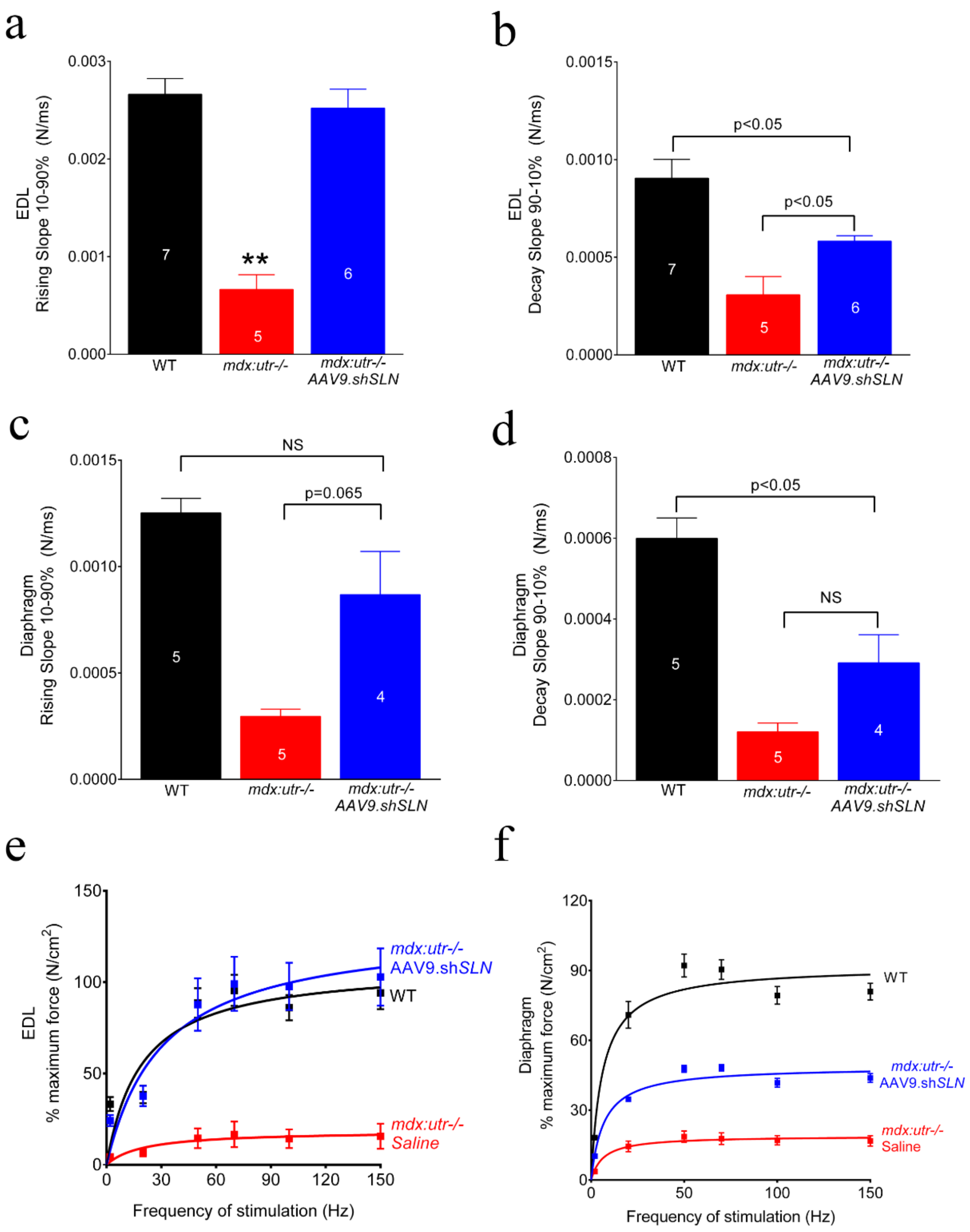

Supplementary Figure 7: AAV9.sh*SLN* treatment improves muscle function in DMD mice. (a, b) The EDL from AAV treated mice exhibits increased rate of contraction as represented by 10%-90% rising slope and the rate of relaxation as represented by 90%-10% decay slope at 2 Hz in comparison with that of *mdx:utr-/-* mice. (c, d) These changes were less prominent in the diaphragm of AAV treated *mdx:utr-/-* mice. The *n* number for each group is shown within the bar. **significantly different from other groups (p<0.005, *t*-test with Welch's correction). (e, f) Force-frequency relationship curve indicates that muscle force is significantly improved at all frequencies in both EDL and diaphragm of AAV treated *mdx:utr-/-* mice. Data are presented as mean ± SEM.

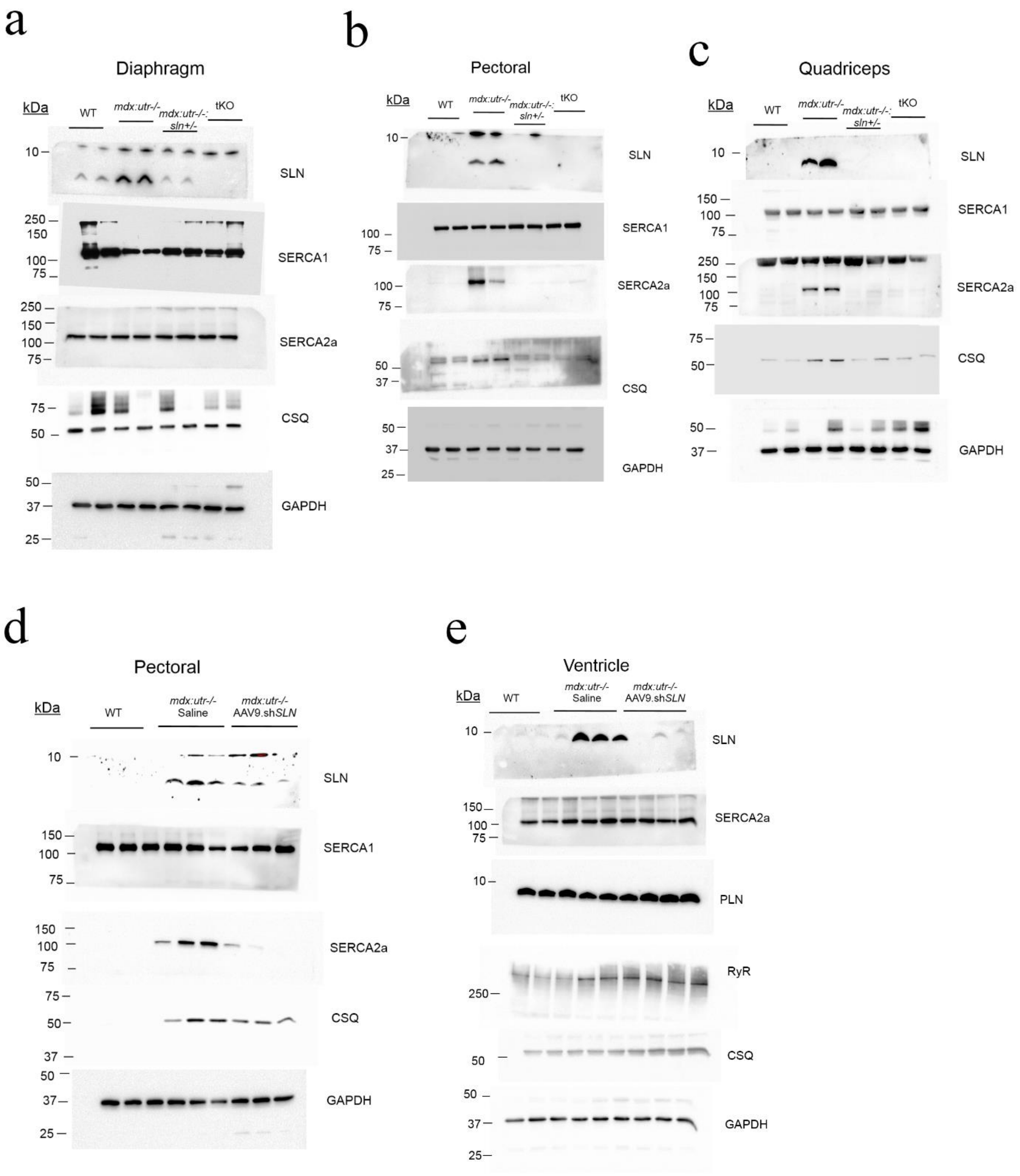

Supplementary Figure 8: Uncropped western images. Western images are corresponding to the images shown in (a) Fig. 1f, (b) Fig. 1g, (c) Fig. 1h, (d) Fig. 6a, and (e) Fig. 6b.

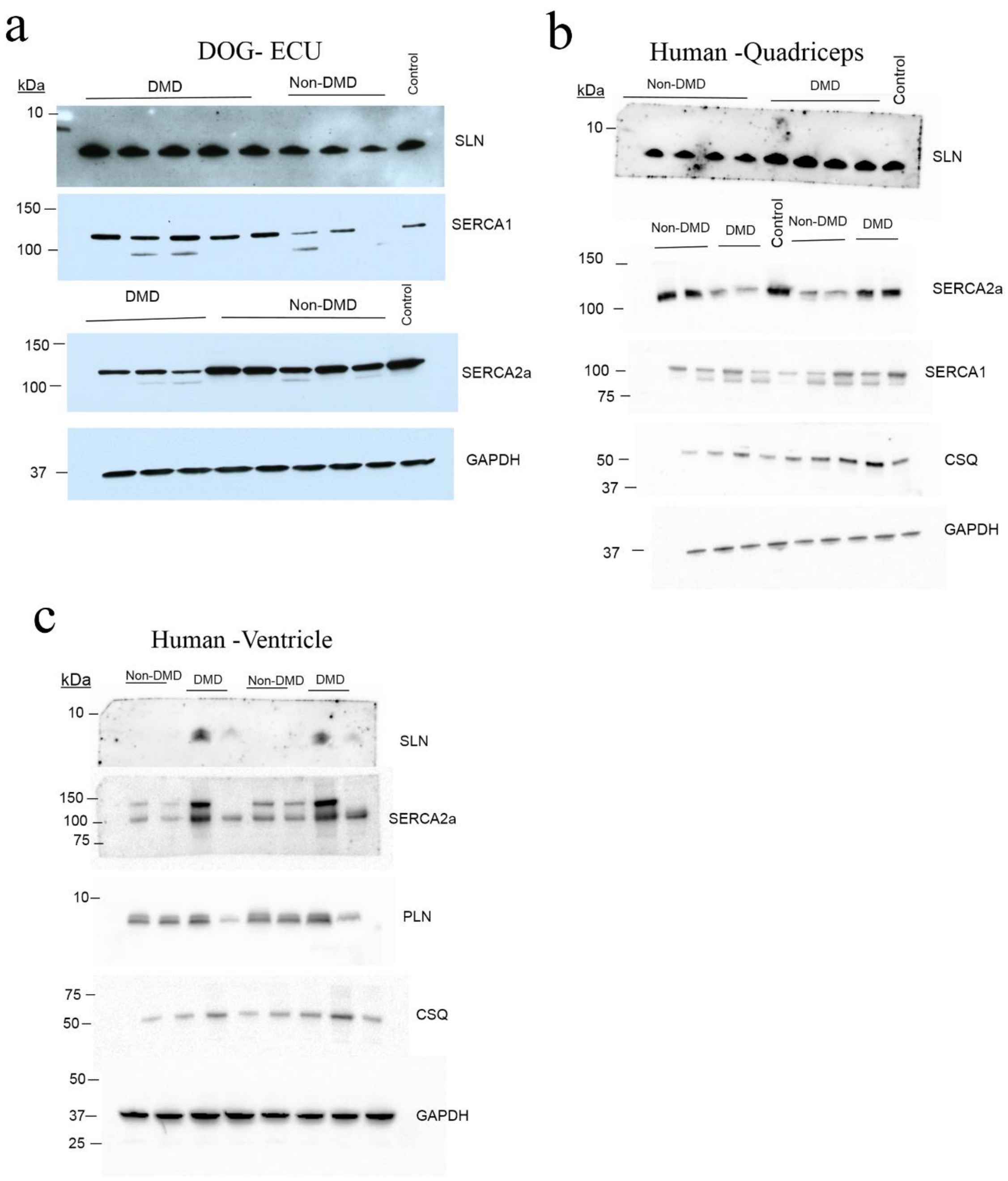

Supplementary Figure 9: Uncropped western images. Western images are corresponding to the images shown in (a) Supplementary Fig. 2a, (b) Fig. 3a, and (c) Fig. 3c.
